# Insulin Resistance in PCOS Patients Enhances Oxidative Stress and Leukocyte Adhesion: Role of Myeloperoxidase

**DOI:** 10.1371/journal.pone.0151960

**Published:** 2016-03-23

**Authors:** Victor M. Victor, Susana Rovira-Llopis, Celia Bañuls, Noelia Diaz-Morales, Arantxa Martinez de Marañon, Cesar Rios-Navarro, Angeles Alvarez, Marcelino Gomez, Milagros Rocha, Antonio Hernández-Mijares

**Affiliations:** 1 Service of Endocrinology, University Hospital Doctor Peset, Foundation for the Promotion of Health and Biomedical Research in the Valencian Region (FISABIO), Valencia, Spain; 2 Institute of Health Research INCLIVA, University of Valencia, Valencia, Spain; 3 CIBERehd - Department of Pharmacology and Physiology, University of Valencia, Valencia, Spain; 4 Department of Physiology, University of Valencia, Valencia, Spain; 5 General Foundation of the University of Valencia, Valencia, Spain; 6 Department of Medicine, University of Valencia, Valencia, Spain; University of Chinese Academy of Sciences, CHINA

## Abstract

Cardiovascular diseases and oxidative stress are related to polycystic ovary syndrome (PCOS) and insulin resistance (IR). We have evaluated the relationship between myeloperoxidase (MPO) and leukocyte activation in PCOS patients according to homeostatic model assessment of IR (HOMA-IR), and have explored a possible correlation between these factors and endocrine and inflammatory parameters. This was a prospective controlled study conducted in an academic medical center. The study population consisted of 101 PCOS subjects and 105 control subjects. We divided PCOS subjects into PCOS non-IR (HOMA-IR<2.5) and PCOS IR (HOMA-IR>2.5). Metabolic and anthropometric parameters, total and mitochondrial reactive oxygen species (ROS) production, MPO levels, interactions between human umbilical vein endothelial cells and leukocytes, adhesion molecules (E-selectin, ICAM-1 and VCAM-1) and proinflammatory cytokines (IL-6 and TNF-α) were evaluated. Oxidative stress was observed in PCOS patients, in whom there was an increase in total and mitochondrial ROS production and MPO levels. Enhanced rolling flux and adhesion, and a decrease in polymorphonuclear cell rolling velocity were also detected in PCOS subjects. Increases in IL-6 and TNF-α and adhesion molecules (E-selectin, ICAM-1 and VCAM-1) were also observed, particularly in the PCOS IR group, providing evidence that inflammation and oxidative stress are related in PCOS patients. HOMA-IR was positively correlated with hsCRP (p<0.001, r = 0.304), ROS production (p<0.01, r = 0.593), leukocyte rolling flux (p<0.05, r = 0.446), E-selectin (p<0.01, r = 0.436) and IL-6 (p<0.001, r = 0.443). The results show an increase in the rate of ROS and MPO levels in PCOS patients in general, and particularly in those with IR. Inflammation in PCOS induces leukocyte-endothelium interactions and a simultaneous increase in IL-6, TNF-α, E-selectin, ICAM-1 and VCAM-1. These conditions are aggravated by the presence of IR.

## Introduction

Polycystic ovary syndrome (PCOS) occurs in 6–20% of reproductive aged women [1–2]. Insulin resistance (IR) is related to PCOS [3], and metabolic syndrome is reported in PCOS patients, thus increasing the risk of major cardiovascular events, morbidity and diabetes and affecting patient quality of life and overall health care costs [4–6].

Inflammation and oxidative stress have been related to the pathogenesis of PCOS [7–8], including an increase in reactive oxygen species (ROS) production by peripheral blood leukocytes [9–10], activation of leukocyte-endothelium interactions [11] and the proinflammatory transcription factor nuclear κβ (NF-κβ), and a rise in the levels of proinflammatory cytokines [12] and C-reactive proteins [13]. Oxidative stress has been implicated in the etiology of IR in leukocytes from PCOS patients, and an increase in leukocytes has been highlighted as a putative marker of low-grade chronic inflammation and early cardiovascular risk in these subjects [13].

In this sense, it has been suggested that some enzymes, such as myeloperoxidase (MPO), a heme protein derived from leukocytes, play an important role in leukocyte-mediated endothelium damage in inflammation and cardiovascular diseases [14]. MPO is released from activated leukocytes at inflammatory sites, generating reactive oxygen species (ROS). However, the antimicrobial activity of MPO can also produce oxidative damage in the endothelium and vessel wall, thus promoting CVD and clinical complications [15]. In light of this, several studies have explored the relationship between MPO and PCOS [16–18].

Leukocytes can adhere to the endothelium and migrate to the bacterial focus, where the pathogen is killed by ROS production and phagocytosis. Under some situations of IR, such as type 2 diabetes, there is an increased recruitment of leukocytes [19] that is associated with endothelial dysfunction. In this sense, arterial stiffness can be more pronounced in PCOS subjects, independently of age, body mass index (BMI) and blood pressure [20]. PCOS is usually linked to endothelial dysfunction due to the high levels of glucose present in patients, and this process is normally associated with endothelial impairment and, in turn, leukocyte-endothelium interactions.

The primary outcome of this study was to evaluate the relationship between MPO levels, ROS production and leukocyte-endothelium interactions, adhesion molecules (E-Selectin, ICAM-1 and VCAM-1) and proinflammatory cytokines (IL-6 and TNF-α) in PCOS according to HOMA-IR. A secondary objective was to assess potential correlations between these factors and endocrine and inflammatory parameters.

## Materials and Methods

### Subjects

The study was conducted in the Service of Endocrinology, University Hospital Dr. Peset, Valencia, Spain. 101 women with PCOS (age 25.8 ± 5.3) and 105 controls (age 26.4 ± 5.6) were selected according to age and BMI (Table 1). Controls were volunteers recruited from the University Hospital Dr Peset and the Faculty of Medicine (University of Valencia). PCOS subjects were diagnosed using the Rotterdam criteria [21], which are as follows: oligoovulation (cycles longer than 35 days or less than 26 days) [22]; elevated free testosterone levels (>0.5 ng/dl; the cut-off level for free testosterone was the mean ± 2 SD according to normal levels in controls); hirsutism (total Ferriman-Gallwey score>7) and polycystic ovaries, identified by transvaginal ultrasonography and following the Rotterdam criteria; i.e. the presence of 12 or more small (2 to 9 mm) follicles in each ovary. Ultrasound scans were performed and scored independently by one of two experienced and blinded reviewers. None of the subjects had any systemic or endocrine disease or galactorrhea, or any condition which could have affected her reproductive physiology. Absence during the previous semester of any medication that might have affected the hypothalamic-pituitary-gonadal axis was confirmed in all subjects.

**Table 1:** Anthropometric parameters, lipoprotein profile, hydrocarbonated metabolism parameters and circulating androgens of PCOS women and control subjects.

	Controls (n = 105)	PCOS HOMA-IR<2.5 (n = 65)	PCOS HOMA-IR≥2.5 (n = 36)	P-value	P-value BMI adjusted
**Age (years)**	26.4 ± 5.6	25.3 ± 5.3	26.6 ± 5.9	0.671	0.249
**Body weight (kg)**	68.4 ± 18.5	68.9 ± 11.7	78.8 ± 10.8	0.144	---
**BMI (kg/m**^**2**^**)**	25.6 ± 6.8	25.4 ± 4.2	28.3 ± 4.6	0.368	---
**Waist (cm)**	82.1 ± 15.7	84.8 ± 10.2	91.1 ± 9.8	0.093	0.155
**Systolic BP (mmHg)**	110.3 ± 14.9	111.6 ± 13.7	111.2 ± 11.5	0.859	0.993
**Diastolic BP (mmHg)**	67.6 ± 12.0	71.0 ± 9.3	75.9 ± 11.6	0.016	0.038
**Total cholesterol (mg/dl)**	173.3 ± 31.3	173.0 ± 33.6	198.3 ± 37.8*	0.011	0.442
**LDLc (mg/dl)**	104.0 ± 24.3	106.1 ± 30.0	124.7 ± 26.2*	0.007	0.412
**HDLc (mg/dl)**	55.7 ± 12.9	53.8 ± 10.7	44.8 ± 13.3*	0.002	0.061
**Triglycerides (mg/dl)**	70.6 ± 36.3	65.6 ± 30.3	143.8 ± 97.5^#^ *	<0.001	0.001
**Apo AI (mg/dl)**	152.9 ± 37.1	148.1 ± 22.6	135.3 ± 27.9	0.108	0.316
**Apo B (mg/dl)**	77.0 ± 19.3	76.3 ± 23.9	96.4 ± 28.1*	0.004	0.090
**hsCRP (mg/l)**	2.07 ± 2.46	3.29 ± 3.42	3.72 ± 3.19*	0.018	0.137
**Glucose (mg/dl)**	83.1 ± 10.5	81.5 ± 8.9	85.6 ± 7.8	0.280	0.483
**Insulin (μIU/ml)**	6.86 ± 2.75	7.23 ± 2.31	19.65 ± 9.12^#^ *	<0.001	<0.001
**HOMA-IR**	1.38 ± 0.56	1.75 ± 0.34	4.17 ± 2.04^#^ *	<0.001	<0.001
**HbA1c (%)**	5.16±0.27	5.19±0.21	5.26±0.28	0.444	0.873
**FSH (mIU/ml)**	4.31 ± 2.36	4.50 ± 1.36	4.59 ± 1.37	0.871	0.503
**LH (mIU/ml)**	4.59 ± 4.04	6.43 ± 4.29*	5.14 ± 2.95	0.015	0.030
**Testosterone (ng/ml)**	0.45 ± 0.21	0.77 ± 0.44*	0.68 ± 0.46*	<0.001	<0.001
**DHEA-S (μg/dl)**	244.7 ± 101.8	331.9 ± 161.1*	273.4 ± 145.3	0.005	0.011
**Androstendione (ng/ml)**	2.79 ± 1.22	4.05 ± 1.88*	4.16 ± 2.24*	<0.001	<0.001
**SHBG (nmol/l)**	106.5 ± 75.4	63.6 ± 52.3*	47.4 ± 50.8*	<0.001	<0.001

Data are expressed as mean ± SD. Statistical significance (p<0.05) was considered when compared by an ANOVA followed by a post hoc test and using BMI as covariate

(* p<0.05 when comparing PCOS vs Controls; # p<0.05 when comparing PCOS HOMA-IR≥2.5 vs HOMA-IR<2.5)

Exclusion criteria were malignant neoplasia, anemia, active infectious diseases or thromboembolism, stroke or history of ischaemic heart disease, diabetes mellitus and the taking of lipid-lowering or antihypertensive drugs.

### Biochemical determinations

An anthropometric and analytical evaluation was performed and weight (kg), height (m) and waist (cm) measured in all subjects. Body mass index (BMI = weight (kg) / height (m)^2^) was then calculated. Blood was collected from the antecubital vein at 8–10 a.m, after 12 hours of fasting, during the follicular phase, on the second/third day of the menstrual cycle or after 3 months of amenorrhea. In cases of very irregular cycles, blood was collected after progesterone deprivation.

Low density lipoproteins cholesterol (LDLc) concentration was calculated using the Friedewald method. Total cholesterol and triglycerides were measured by means of enzymatic assays, and high density lipoproteins cholesterol (HDLc) concentrations were recorded with a Beckman LX-20 autoanalyser (Beckman Coulter, La Brea, CA, USA) using a direct method. The intraserial variation coefficient was <3.5% for all determinations. HbA1c was determined with an Automatic Glycohemoglobin Analyzer (Arkray, Inc., 73 KYOTO, Japan). Apolipoprotein (Apo) AI and B were determined by immunonephelometry (Dade Behring BNII, Marburg, Germany) with an intra-assay variation coefficient of <5.5%. High sensitivity C-reactive protein (hsCRP) was evaluated by an immunonephelometric assay (Behring Nephelometer II, Dade Behring, Inc., Newark, DE, USA) with an intra-assay coefficient of variation of 8.7% and sensitivity of 0.01 mg/L. Glucose levels were measured using enzymatic techniques and a Dax-72 autoanalyzer (Bayer Diagnostic, Tarrytown, New York, USA). The enzymatic luminescence technique was employed to measure insulin. Samples were processed immediately in order to avoid haemolysis and were frozen until analysis. IR was calculated by homeostasis model assessment (HOMA) using baseline glucose and insulin: HOMA = (fasting insulin (μU/ml) × fasting glucose (mmol/L)/22.5. PCOS patients were classified as PCOS-IR when the HOMA index was > than 2.5 and PCOS non-IR when the HOMA index was <2.5, as in a previous study [23]. This cut-off point for IR has been established in our hospital’s Endocrinology Department based on the distribution of the HOMA index for women in our clinical setting (University Hospital Dr. Peset, Valencia, Spain).

Serum luteinizing hormone (LH), follicle-stimulating hormone (FSH) were measured using a 2-site monoclonal non-isotopic system (Architect, Abbott Laboratories, Abbott Park, IL). Sex hormone binding globulin (SHBG), androstendione and testosterone were measured using specialised chemiluminiscence techniques in our hospital’s Clinical Analysis Service. Dehydroepiandrosterone-sulfate (DHEAS) was measured using a specific chemiluminescence technique.

Insulin resistance, medication and a documented history of vascular disease (ischemic cardiopathy, peripheral arteriopathy, or cerebrovascular accident) or diabetes were ruled out in all the volunteer control subjects. Umbilical cords were obtained from control women during normal delivery, and those damaged by hematoma or under pathological conditions were discarded. Informed written consent was obtained from all subjects prior to participation. The study was approved by the ethics committee of the University Hospital Dr. Peset and was performed in accordance with the Helsinki declaration.

### Cells

Human polymononuclear leukocytes (PMNs) were obtained from citrated blood samples and incubated for 45 min with dextran (3%). The supernatant was centrifuged at 250g for 25 min over Fycoll-Hypaque. Lysis buffer was added to the pellet and centrifuged at room temperature (100g, 5 min). PMNs were evaluated in a Scepter device (Millipore, MA, USA), washed in HBSS medium and stored in complete RPMI media.

#### Measurement of total and mitochondrial ROS production

Total ROS production was evaluated in PMNs by two methods. Cells were incubated (30 min) with the fluorescent probe (5 x 10^−6^ mol/L) 2’,7’-dichlorodihydrofluorescein diacetate (DCFH-DA) [24]. First, ROS production was assessed by fluorimetry using a Synergy Mx plate reader (BioTek Instruments, Winooski, VT). Second, it was assessed using a fluorescence microscope (IX81, Olympus, Hamburg, Germany) coupled with the static cytometry software ‘ScanR’ version 2.03.2 (Olympus). For static cytometry, PMNs from each subject were seeded in triplicate in 48-well plates and 16 images per well were recorded and analyzed [24].

The fluorescent probe Mitosox Red (5μM) was employed to assess mitochondrial ROS production. Leukocytes were seeded in 48-well plates and incubated for 30 min with the respective fluorochrome and washed with HBSS. 16 images per well were recorded with an IX81 Olympus fluorescence microscope (Olympus, Hamburg, Germany), and the static cytometry software ‘ScanR’ version 2.03.2 (Olympus) was used to quantify fluorescence individually (per cell). Fluorescent probes were purchased from Invitrogen (Life Technologies, Barcelona, Spain).

### MPO Assay

Plasma MPO concentrations were measured using an immunoassay based on a double-antibody ‘sandwich’ technique according to the manufacturer's instructions (MPO EIA kit, Cayman Chemical) [14]. This is an immunometric assay in which a monoclonal antibody specific for MPO is used to capture MPO in the plate wells and an HRP-labeled MPO monoclonal antibody is employed to detect the captured MPO. The concentration of the enzyme is determined using the chromogenic substrate for HRP, 3,3’,5,5’-tetramethylbenzidine. The intra-assay coefficient of variation (CV) of this immunoassay is 6.7% and the inter-assay CV is 8.3%.

### Adhesion assay

The human umbilical vein endothelial cells (HUVEC) used in the adhesion studies were harvested from umbilical cords by treating them with collagenase. In short, umbilical cord veins were rinsed of blood products with warm phosphate-buffered saline (PBS), after which the vein was filled with collagenase (1 mg/mL) for 17 min at 37°C. The cords were then gently massaged to ensure detachment of endothelial cells from the vessel wall. The digest was collected, centrifuged and pelleted. The pellet was resuspended in endothelial cell growth medium (EGM-2) inside T25 culture flasks in which cells were cultured until confluence. After reaching confluence, primary cultures were detached with trypsin and transferred to 6-well plate culture dishes. HUVEC were cultured on fibronectin (5 mg/mL)-coated 25-mm plastic coverslips until confluent (≈48 h). For the flow chamber in vitro model study, human leukocytes (1 x 10^6^ cells/mL) were resuspended in Dulbecco’s PBS containing 20 x 10^−3^ mol/L HEPES and 0.1% human serum albumin, and were drawn across the HUVEC monolayer under a flow rate of 0.36 mL/min (approximately shear stress of 0.7 dyne/cm^2^) under a microscope (Nikon Eclipse TE 2000-S; Amstleveen, The Netherlands) connected to a video camera (Sony Exware HAD; Koeln, Germany) [25]. Images in a single field of view were recorded over a 5 min period and rolling and adhesion parameters were evaluated. The rolling velocity in the field of focus was determined by measuring the time required by 20 consecutive leukocytes to cover a distance of 100 μm. Leukocyte rolling flux was estimated as the number of leukocytes rolling over 100 μm^2^ of the endothelial monolayer during a 1 min period. Adhesion was evaluated by counting the number of PMNs that maintained stable contact with HUVEC for 30 sec. Platelet-activating factor (1 μmol/L, 1 h) and TNF-α (10 ng/mL, 4 h) were used as positive controls for leukocytes and HUVEC, respectively.

### Adhesion molecules and levels TNF-α and IL-6 levels

A Luminex 200 flow analyser system was employed to analyse adhesion molecules (E-selectin, ICAM-1 and VCAM-1) and proinflammatory cytokines (IL-6 and TNF-α) in serum from controls and PCOS patients (Austin, TX, USA).

### Drugs and solutions

Kits for glucose, insulin, total cholesterol, triglycerides, HDLc and LDLc were purchased from Abbott Laboratories (Abbott Park, IL, USA). HsCRP kits were supplied by Beckman Corp (Brea, A, USA). The HbA1c kit was purchased from Menarini Diagnostics (Florence, Italy). The MPO kit was purchased from Cayman Chemical (Michigan, USA).

Glucose, trypan blue, arginine, glutathione reductase, H_2_O_2_, haemoglobin, RPMI1640 supplemented with 20 mM HEPES, HBSS, TNF-α, human serum albumin (HSA, Albuminate 25%) and fibronectin were obtained from Sigma-Aldrich (Sigma Chem. Co., St. Louis, MO, USA). Dextran was acquired from Fluka (St. Louis, MO, USA). HBSS was supplied by Cambrex (Verviers, Belgium). DCFH-DA and Mitosox tracker were provided by Calbiochem (San Diego, CA, USA). Dulbecco’s PBS—with (DPBS^+^) or without (DPBS^-^) Ca^2+^ and Mg^2+^—endothelial cell growth medium culture media and fetal bovine serum were obtained from LONZA (Verviers, Belgium). Plastic coverslips (diameter of 25 mm) were purchased from Nunc (Thermo Fisher Scientific). PBS, collagenase, and trypsin-EDTA were obtained from Invitrogen (Eugene, OR, USA). Ficoll-Paque TM Plus was purchased from GE Healthcare (Little Chalfont, Buckinghamshire,UK).

### Statistical analysis

Data analysis was performed with SPSS 17.0. The values in Table 1 are mean ± SD. Bar graphs show mean ± SEM. Data were compared with a one-way analysis of variance (ANOVA), followed by a post hoc test. Analysis of covariance was employed to minimize the potential influence of BMI. Serum lipid and biochemical parameter changes were analyzed, using BMI as a covariate. Correlations were calculated using Pearson’s correlation coefficient. Significant differences were considered when p<0.05.

## Results

### Metabolic and clinical characteristics

Baseline anthropometric characteristics of the PCOS and control groups were found to be similar, with no statistically significant differences regarding age, body weight, BMI, waist circumference or systolic blood pressure, as shown in Table 1. However, as expected, there was a trend towards higher BMI levels in PCOS women as their insulin resistance increased. There was a significant increase in LH, testosterone, DHEA-S and androstendione levels and a decrease in SHBG in patients with HOMA-IR<2.5 with respect to controls. Furthermore, patients with HOMA-IR>2.5 exhibited a significant increase in diastolic BP, total cholesterol, LDLc, triglycerides, hsCRP, insulin, testosterone and androstendione (p<0.05 for all) and a significant reduction in HDLc and SHBG (p<0.05) with respect to the control group (Table 1). These differences remained after adjustment for BMI, with the exception of diastolic BP, total cholesterol, LDLc, HDLc, Apolipoprotein B and hsCRP. No changes were detected in the biochemical parameters of any of the PCOS groups according to HOMA-IR, exception of triglycerides and insulin (p<0.05).

The prevalence of MetS was 38.9%, 4.6% and 7.9% among PCOS IR, non-IR and controls, respectively (p<0.001). When metabolic and oxidative stress parameters and MPO were analyzed according to presence or absence of MetS in PCOS, we observed that patients with MetS had an altered metabolic profile and higher levels of ROS production, hsCRP and ICAM-1 (S1 Table).

### Total and mitochondrial ROS production

**Fig 1:**
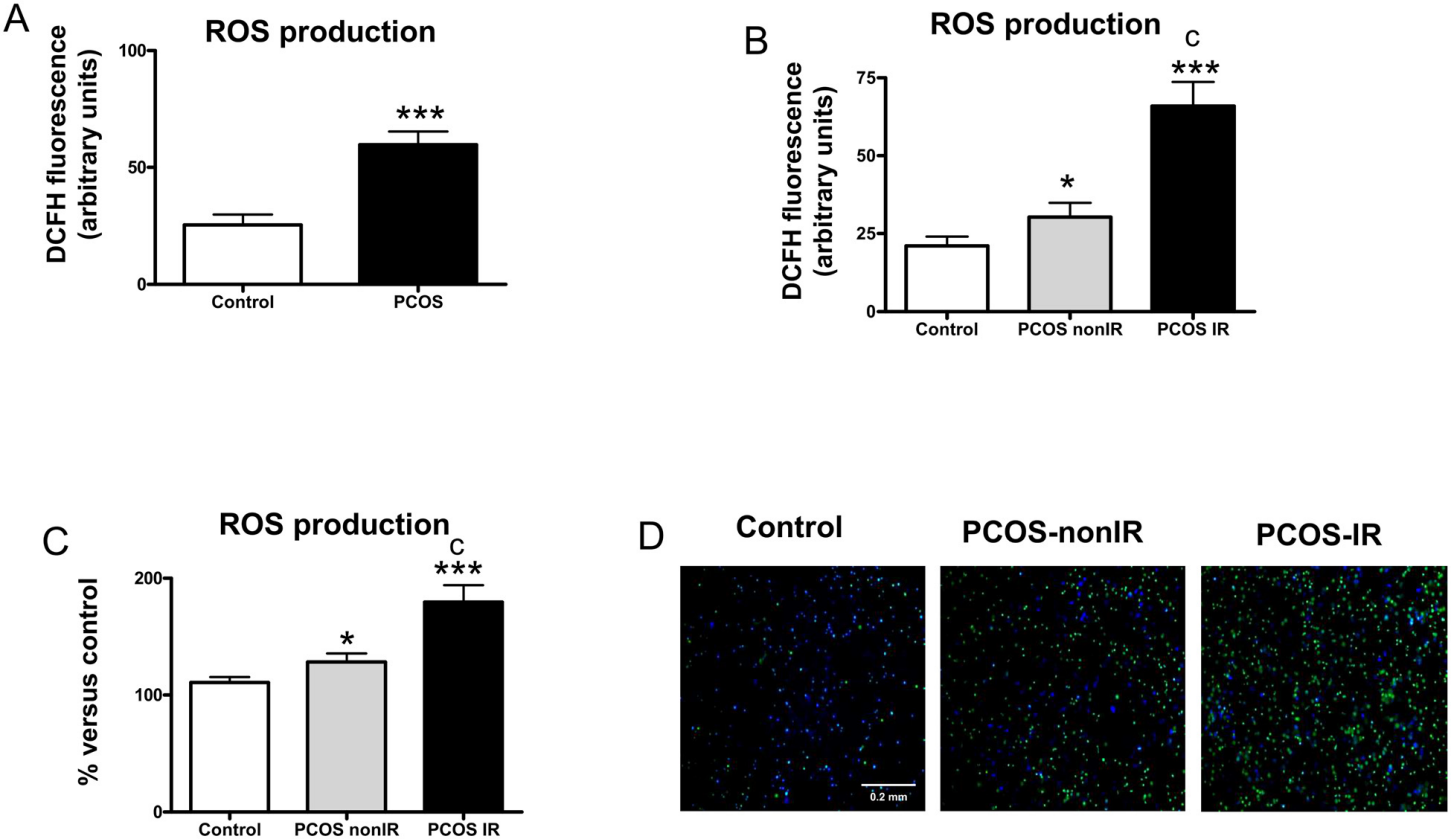
Effects of PCOS on levels of ROS in PMN. (A) Levels of DCFH-DA fluorescence measured by fluorimetry in controls and PCOS subjects; (B) Levels of DCFH fluorescence in controls and in PCOS non-IR and PCOS IR subjects; (C) Mean DCFH fluorescence assessed by static cytometry; % vs control. (D) Representative images of DCFH-DA fluorescence in PMNs assessed by fluorescence microscopy; nuclei: Hoechst 33342 signal (blue); ROS: DCFH-DA signal (green). *p<0.05 ***p<0.001 vs. Control; ^c^p<0.001 between PCOS non-IR and PCOS IR subjects.

DCFH-DA fluorescence was significantly higher (p<0.001) in leukocytes from PCOS patients (Fig 1A). When PCOS subjects were divided into insulin-resistant (PCOS IR group, HOMA-IR>2.5) and non-insulin-resistant (PCOS non-IR group, HOMA-IR<2.5) groups, an increase in DCFH fluorescence with respect to controls was observed in the PCOS non-IR (p<0.05, Fig 1B and 1C) and PCOS IR (p<0.001, Fig 1B and 1C) groups, which was evidence of enhanced ROS production and, consequently, oxidative stress. ROS production (p<0.001, Fig 1B and 1C) was significantly higher in the PCOS IR group than in the PCOS non-IR group. Representative images of fluorescence microscopy are shown in Fig 1D.

In addition, we evaluated levels of ROS depending on the levels of glucose and insulin in each PCOS patient, finding a significant correlation between ROS production and HOMA (r = 0.621, p = 0.002), insulin (r = 0.581, p = 0.004) and glucose (r = 0.301, p = 0.163). Moreover, when we subdivided the populations of PCOS patients according to tertiles of insulin (T1< 7.6, T2: 7.6–15.6, T3 >15.6 μUI/ml), we observed that those with a higher tertile of insulin presented higher levels of ROS production (T1 = 34.5±19.4, T2 = 36.4±22.5, T3 = 62.6±12.9; p = 0.017), and when we subdivided them according to tertiles of glucose (T1< 81.0, T2: 81.0–88.0, T3 >88.0 mg/dl), we observed that those with the higher tertile of glucose presented an increase of ROS production (T1 = 45.9±23.8, T2 = 34.9±19.6, T3 = 61.2±15.2; p<0.05).

**Fig 2:**
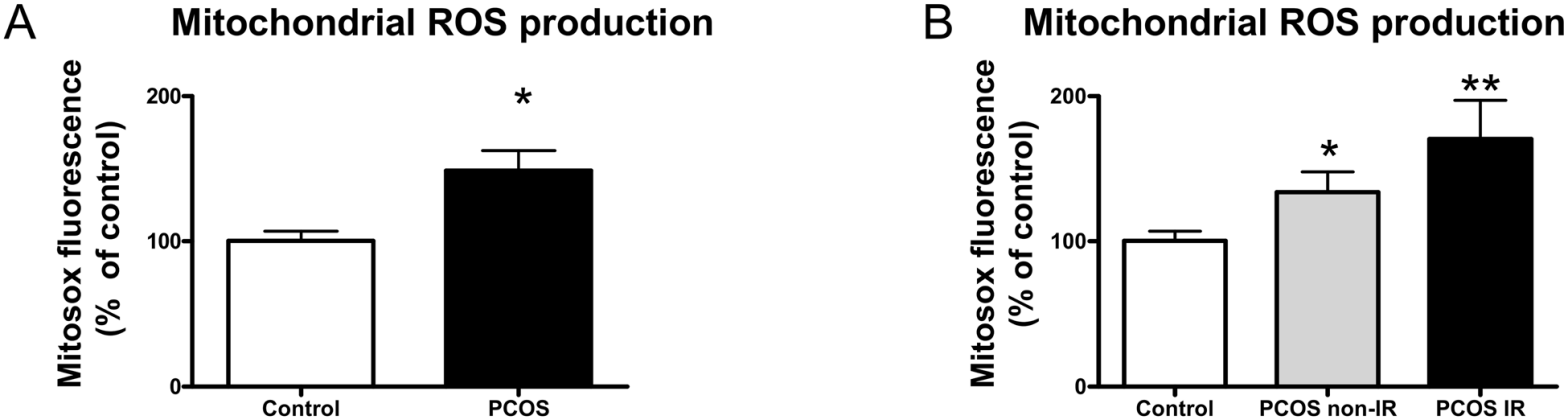
Leukocyte mitochondrial ROS production in PCOS non-IR and IR patients and control women. A) Mitochondrial ROS (Mitosox Red Fluorescence) in PCOS versus control subjects. B) Mitochondrial ROS (Mitosox Red Fluorescence) in PCOS non IR and IR patients versus control subjects. Values in the bar graphs were obtained by calculating the percentage of fluorescence intensity relative to the control. *p<0.05 and **p<0.01 PCOS versus control group.

Mitosox Red fluorescence was significantly higher (p<0.05) in leukocytes from PCOS patients (Fig 2A). When PCOS subjects were divided into insulin-resistant (PCOS IR group, HOMA-IR>2.5) and non-insulin-resistant (PCOS non-IR group, HOMA-IR<2.5) groups, an increase in Mitosox Red fluorescence with respect to controls was observed in the PCOS non-IR (p<0.05, Fig 2B) and PCOS IR (p<0.05, Fig 2B) groups, which was evidence of enhanced mitochondrial ROS production, thus indicating conditions of oxidative stress.

### Myeloperoxidase levels

**Fig 3:**
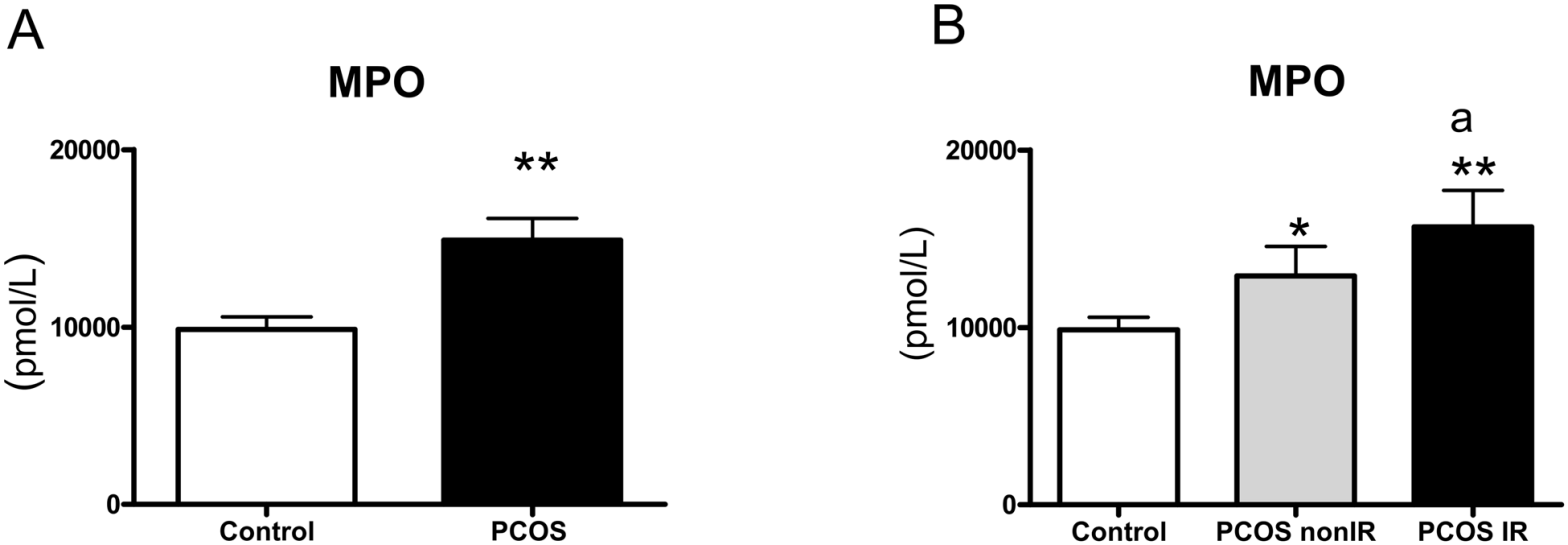
Plasma MPO concentrations in PCOS and control subjects (A) and in control, PCOS non-IR and PCOS IR subjects (B). *p<0.05 and **p<0.01 vs. Control; ^a^p<0.05 between PCOS non-IR and PCOS IR subjects.

Therefore, we evaluated the levels of MPO in plasma from PCOS patients and controls and found that the levels of MPO were higher among the former (p<0.01, Fig 3A). In addition, we assessed MPO levels according to the presence or absence of IR and found them to be significantly higher in the PCOS IR (p<0.01) and non-IR PCOS (p<0.05) groups than in controls (Fig 3B). Levels of MPO were higher (p<0.05) in the PCOS IR group than in the PCOS non-IR group.

### Leukocyte-endothelial interactions

**Fig 4:**
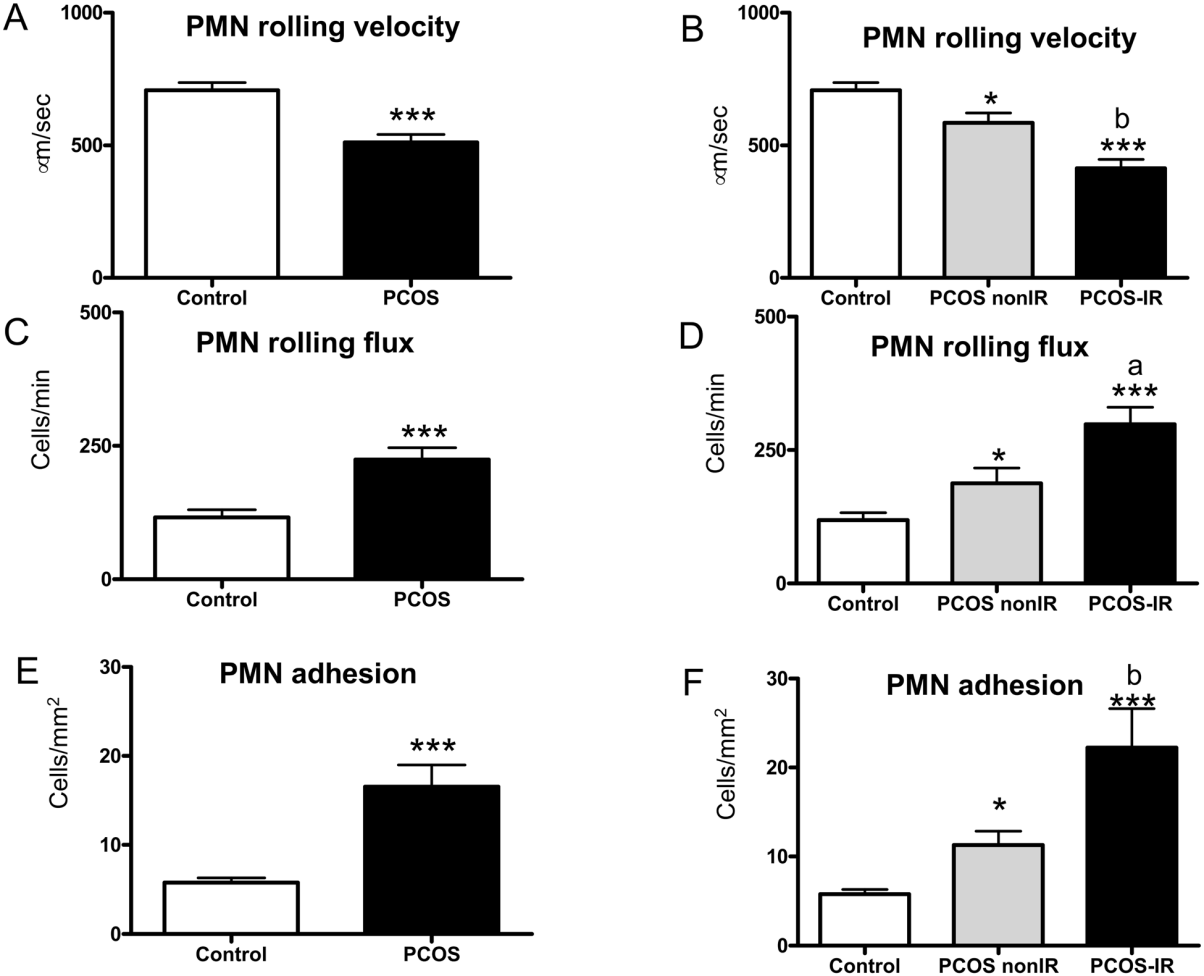
Leukocyte/endothelium interactions in PCOS and control subjects. PMN rolling velocity (μsecond^-1^) (A), rolling flux (PMN per minute) (C), and PMN adhesion (PMN per square millimetre) (E); and PCOS non-IR and PCOS IR subjects: PMN rolling velocity (μsecond^-1^) (B), rolling flux (PMN per minute) (D), and PMN adhesion (PMN per square millimetre) (F). *p<0.05 and ***p<0.001 vs. Control. ^a^p<0.05 and ^b^p<0.01 between PCOS non-IR and PCOS IR subjects.

We aimed to determine whether the IR control status of PCOS patients is related to leukocyte-endothelial interactions. In PCOS patients, we found a decrease in PMN rolling velocity (Fig 4A, p<0.001) and an increase in PMN rolling flux (Fig 4C, p<0.001) and PMN adhesion (Fig 4E, p<0.001) with respect to controls. Furthermore, when these parameters were evaluated according to the presence or absence of IR, leukocyte rolling velocity was found to be diminished in both PCOS groups dependently of IR control status (p<0.05 in the PCOS non-IR group, p<0.001 in the PCOS IR group, Fig 4B). Leukocyte rolling flux (Fig 4D) was enhanced in non-IR PCOS patients (p<0.05) and was significantly more pronounced in PCOS-IR patients (p<0.001). Leukocyte adhesion to the endothelium was enhanced in both PCOS groups, (p<0.05 in PCOS non-IR patients, and p<0.001 in PCOS-IR patients, Fig 4F) in comparison to controls. In addition, there was a significant decrease (p<0.01, Fig 4B) of PMN rolling velocity and an increase of PMN rolling flux (p<0.05, Fig 4D) and PMN adhesion (p<0.01, Fig 4F) in the PCOS IR group with respect to the PCOS non-IR group.

### Levels of adhesion molecules and proinflammatory cytokines

**Fig 5:**
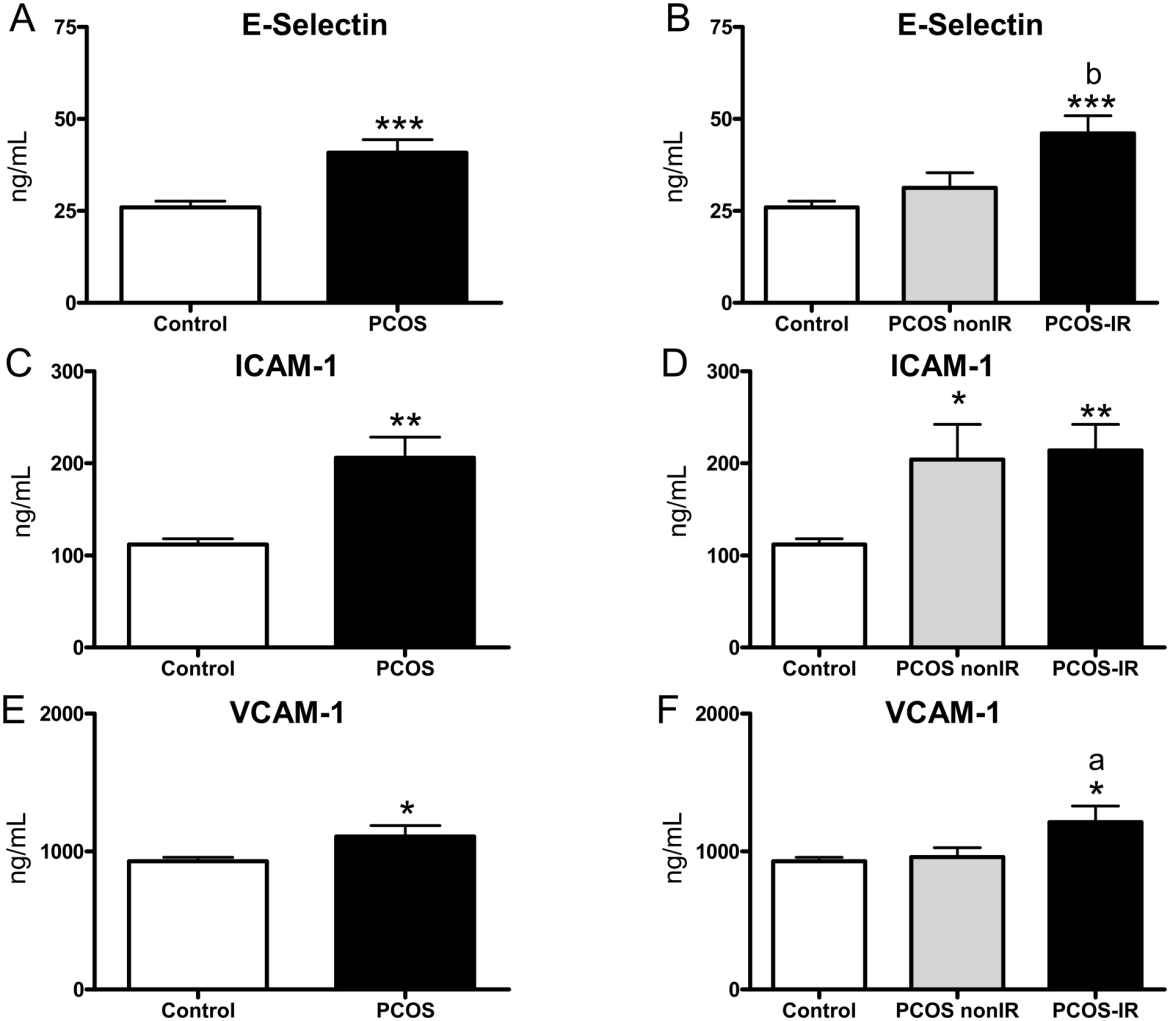
Adhesion molecules in the serum of PCOS and control subjects. (A) E-selectin, (C) ICAM-1 and (E) VCAM-1; and PCOS non-IR, PCOS IR and control subjects. (B) E-selectin, (D) ICAM-1 and (F) VCAM-1. *p<0.05, **p<0.01, ***p<0.001 with respect to the control group; ^a^p<0.05 and ^b^p<0.01 between PCOS non-IR and PCOS IR subjects.

PCOS patients exhibited increased levels of E-Selectin (Fig 5A, p<0.001), ICAM-1 (Fig 5C, p<0.01) and VCAM-1 (Fig 5E, p<0.05). In addition, when these parameters were evaluated according to the presence or absence of IR, an increase in the adhesion molecule ICAM-1 was detected in PCOS non-IR patients (p<0.05, Fig 5D) and an increase in E-selectin (p<0.001, Fig 5B), ICAM-1 (p<0.01, Fig 5D) and VCAM-1 levels was observed in PCOS IR subjects (p<0.05, Fig 5F) with respect to controls. In addition, there was a significant increase in E-selectin (p<0.01) and VCAM-1 (p<0.05) in the PCOS IR group with respect to the non-IR group.

**Fig 6:**
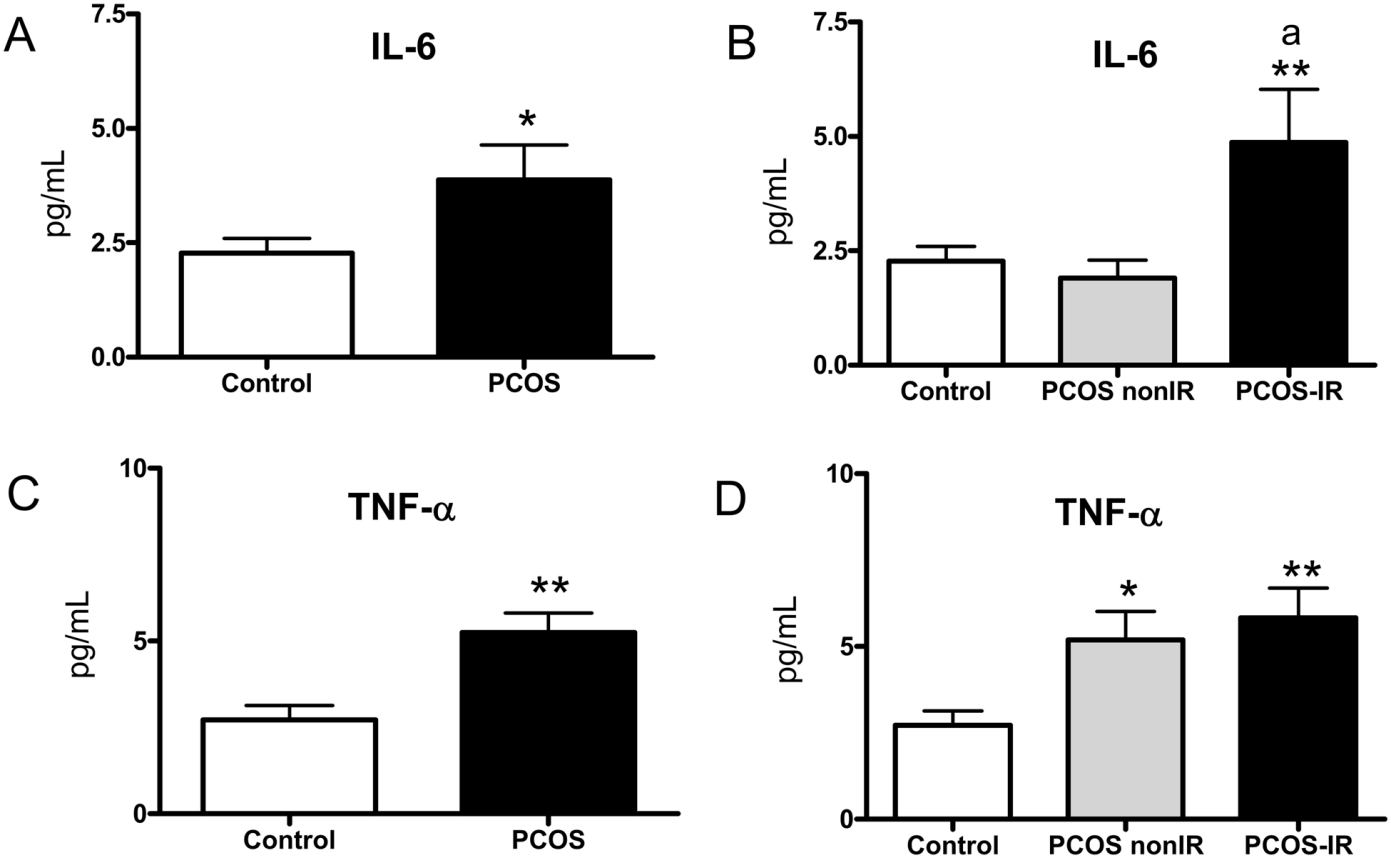
Proinflammatory cytokines in the serum of PCOS and control subjects. (A) IL-6, and (C) TNF-α; and PCOS non-IR, PCOS IR and control subjects. (B) IL-6, and (D) TNF-α. *p<0.05, **p<0.01 with respect to control group; ^a^p<0.05 between PCOS non-IR and PCOS IR subjects.

Finally, we found an increase in the proinflammatory cytokines IL-6 (Fig 6A, p<0.05) and TNF-α (Fig 6C, p<0.01) with respect to controls. These effects were more evident among IR patients (p<0.05 in PCOS non-IR for TNF-α and p<0.01 in IR subjects for IL-6 and TNF-α Fig 6B and 6D). In addition, there was an increase of IL-6 (p<0.05) in the PCOS IR group with respect to the PCOS non-IR group.

### Correlation studies

**Fig 7:**
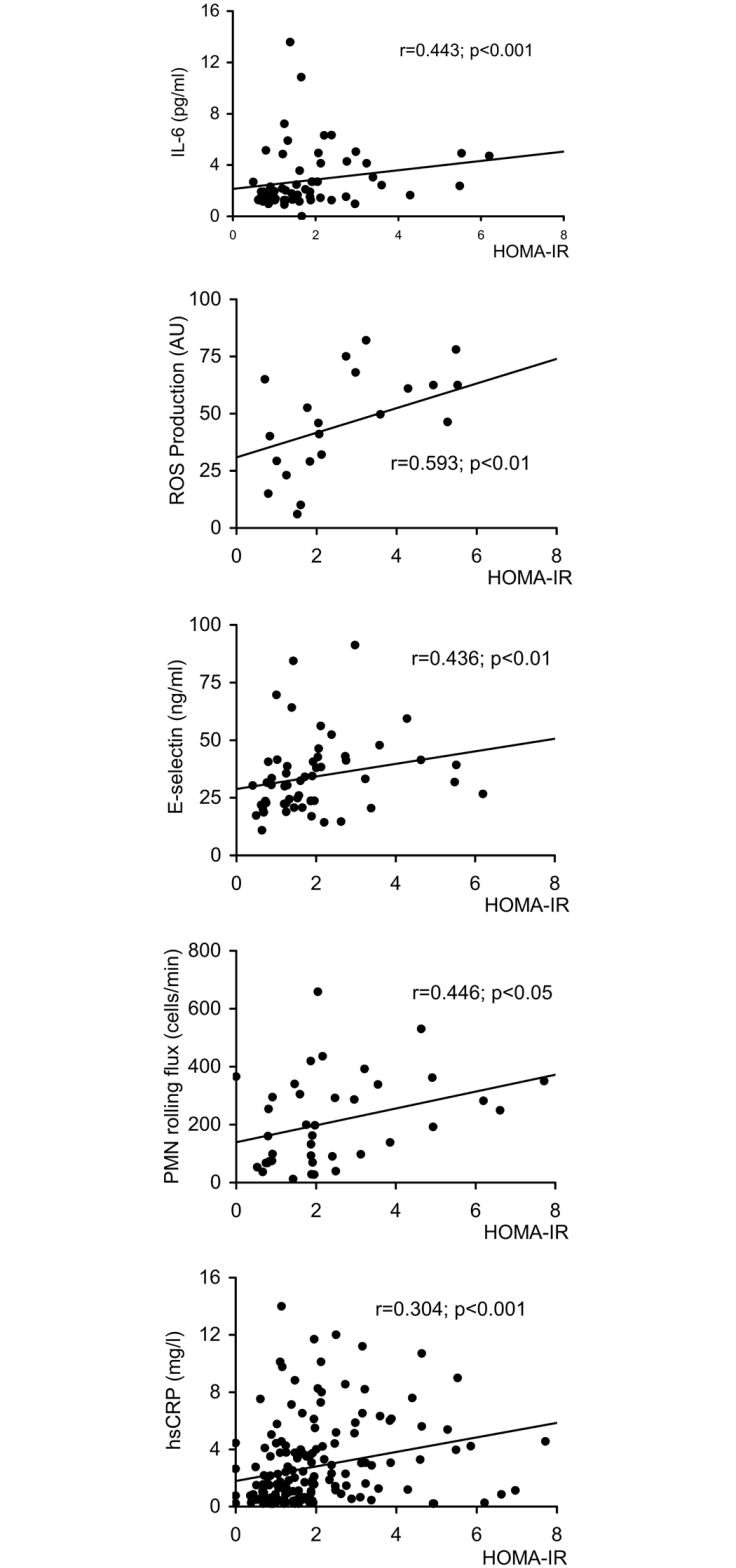
Correlation studies of HOMA-IR and inflammation and adhesion parameters in PCOS women.

When we explored potential correlations among HOMA-IR and inflammation/adhesion parameters we found that the former was positively correlated with hsCRP (p<0.001, r = 0.304), ROS production (p<0.01, r = 0.593), leukocyte rolling flux (p<0.05, r = 0.446), E-selectin (p<0.01, r = 0.436) and IL-6 (p<0.001, r = 0.443) (Fig 7).

## Discussion

It has previously been described that PCOS subjects are in a proinflammatory state [26], and that IR usually can coexist with PCOS, not only in obese patients, but also in lean subjects. Although vascular dysfunction and endothelial/leukocyte interactions are key features of IR [27] and are thought to be a major cause of IR-associated vascular complications, the underlying molecular mechanisms are still not understood. In the present study, we have evaluated, as primary outcome measures, the relationship between MPO, oxidative stress, endothelial/leukocyte interactions and adhesion molecules in PCOS patients with different HOMA-IR indexes, and have explored possible correlations between these factors and endocrine and inflammatory parameters.

PCOS is a heterogeneous syndrome associated with a wide range of endocrine and metabolic abnormalities, including hyperinsulinaemia, hyperglycaemia, dyslipidaemia and obesity, which are regarded as hallmark components of MetS. In our population, the prevalence of MetS in IR vs non-IR PCOS patients was significantly higher, which is consistent with previous reports showing a higher incidence among approximately one-third of PCOS women, especially among those with the highest BMIs and insulin levels [28]. While obesity is regarded as one of the putative factors leading to MetS, the link between PCOS and MetS would seem to be attributable mainly to IR [29].

ROS can play an important role in hyperglycemia-mediated microvascular complications and endothelial dysfunction in IR conditions [30–31]. In fact, it has been demonstrated that PCOS patients have an increased risk of developing metabolic syndrome, which may be related to oxidative stress and cardiovascular events [6]. Our results confirm that PCOS is related to an increase in ROS production in PMN, and show that this ROS production is related to the presence of IR. In the case of MPO, an important leukocyte-derived pro-oxidant enzyme, some studies have focused specifically on its relationship with PCOS or conditions of IR. In the present study, we have observed that levels of MPO are higher in the leukocytes of PCOS patients, and that this increase is more pronounced in the presence of IR. Our results are in accordance with those of Ribeiro et al., who reported increased levels of MPO in IR PCOS patients [16].

MPO seems to play an important role in endothelial dysfunction [32], is released from granules of activated leukocytes, and can generate ROS as a system of defense against bacteria [33]. However, the antimicrobial activity of MPO can also lead to oxidative damage of the endothelium and vessel wall. Activated leukocyte-released MPO binds to the vascular wall for a long time and can release ROS continuously, therefore increasing endothelial damage [15]. In this context, our group has previously demonstrated an increase in MPO levels in diabetic patients that correlated with the presence of nephropathy and leukocyte-endothelium interactions [14], thus pointing to the importance of MPO in conditions of oxidative stress. However, other studies have shown that poor glycemic control in diabetic patients results in decreased histochemical MPO activity in neutrophils [34].

There are several mechanisms by which MPO can promote the atherosclerotic process: oxidation of LDLc [35]; MPO-induced oxidation of HDLc [36]; and consumption and catabolism of endothelial-derived nitric oxide, which can lead to endothelial dysfunction and plaque formation [37–39]. Furthermore, a correlation has been reported between plasma MPO concentration and red blood cell rigidity index in type-2 diabetes patients with coronary heart disease [40]. These findings suggest that MPO functions as a mediator of regulatory mechanism in microcirculation.

Pathophysiological and inflammatory states such as hypertension and atherosclerosis are characterized by leukocyte recruitment to the arterial wall [41]. To study this process, we have used an in vitro model in which human leukocytes flow over a monolayer of human endothelial cells with a shear stress similar to that observed in vivo [11]. This reproduces the process that precedes inflammation in vivo (rolling and adhesion) and which is critical to homeostasis and vascular cell integrity. If these interactions are exacerbated, the vascular dysfunction and injury associated with many CVD can occur.

In the present study, we have demonstrated that the inflammatory state associated with PCOS is exacerbated in PCOS IR patients, in which there is a decrease in PMN rolling velocity and an increase in rolling flux and adhesion, thereby inducing leukocyte-endothelium interactions. In relation to this, we have detected an increase in the adhesion molecule ICAM-1 in PCOS non-IR subjects, and E-selectin, ICAM-1 and VCAM-1 levels in their PCOS IR counterparts. An increase of circulating ICAM-1 has previously been described in PCOS [42]. In addition, there was a significant increase in E-selectin and VCAM-1 (p<0.05) in the PCOS IR group with respect to the non-IR group, which underlines the essential role of IR in increased levels of adhesion molecules. In the case of proinflammatory cytokines, higher levels of these markers were observed in the PCOS patients, particularly in those with IR, thus highlighting the importance of IR in the development of inflammation in PCOS patients.

Finally, we explored potential correlations between HOMA and inflammation/adhesion parameters and found that the former was positively correlated with hsCRP, ROS production, leukocyte rolling flux, E-selectin and IL-6. These results suggest that IR is related to the inflammatory state that underlies PCOS and leads to an increase in MPO activity and ROS production by leukocytes, which, in turn, enhances leukocyte-endothelium interactions and, consequently, cardiovascular complications. In fact, the MPO molecule is thought to be capable of attracting leukocytes to the vascular wall via its electrostatic characteristics [43], which makes it a main player of inflammation [44].

We did not observe variations in HbA1c levels between the different groups of patients and controls, but the evaluation of fasting glucose levels does not necessarily rule out abnormal glucose tolerance in PCOS, which represents a potential limitation of this study.

## Conclusions

In conclusion, we demonstrate an increase in the rate of ROS and in concentrations of MPO in PCOS patients in general, and particularly in those with IR. Inflammation associated with PCOS can induce leukocyte-endothelium interactions and a simultaneous increase in IL-6, TNF-α and the adhesion molecules E-selectin, ICAM-1 and VCAM-1, conditions that are aggravated by the presence of IR. Enhanced levels of ROS and MPO in PCOS, especially in PCOS-IR patients, may represent an important underlying factor in the clinical complications seen in PCOS subjects.

## Supporting Information

S1 Table Metabolic and oxidative parameters in PCOS women according the presence of metabolic syndrome. Data are expressed as mean ± SD. Statistical significance (p<0.05) was considered when compared by an umpaired Student’s t-test.
(DOC)

## Abbreviations

PCOS: polycystic ovary syndrome
IR: insulin resistance
MPO: myeloperoxidase
HOMA: homeostatic model assessment
ROS: reactive oxygen species
ICAM-1: intercellular adhesion molecule 1
VCAM-1: vascular cell adhesion molecule 1
IL-6: interleukin 6
TNF-α: tumor necrosis factor alpha
hsCRP: high sensitivity c-reactive protein
CVD: cardiovascular disease
NF-κβ: nuclear factor kappa B
BMI: body mass index
LDLc: low density lipoproteins cholesterol
HDLc: high density lipoproteins cholesterol
Apo: apolipoprotein; LH, luteinizing hormone
FSH: follicle-stimulating hormone
SHBG: sex hormone binding globulin
DHEAS: dehydroepiandrosterone-sulfate
PMNs: polymorphonuclear leukocytes
DCFH-DA: dichlorodihydrofluorescein diacetate
HUVEC: human umbilical vein endothelial cells
MetS: Metabolic Syndrome
