# Supplementary material for: Insulin Resistance in PCOS Patients Enhances Oxidative Stress and Leukocyte Adhesion: Role of Myeloperoxidase
Source: PLoS One. 2016 Mar 23;11(3):e0151960. doi: 10.1371/journal.pone.0151960 (PMC4805297; doi:10.1371/journal.pone.0151960)
Supplement: S1 Table — Data are expressed as mean ± SD. Statistical significance (p<0.05) was considered when compared by an umpaired Student’s t-test. (DOC) [file pone.0151960.s001.doc]

S1 Table. Metabolic and oxidative parameters in PCOS women according the presence of metabolic syndrome

|  | PCOS without MetS | PCOS with Mets | P-value |
| --- | --- | --- | --- |
| Age (years) | 24.6 ± 4.9 | 30.8 ± 4.1 | <0.001 |
| Body weight (kg) | 68.5 ± 15.3 | 95.1 ± 12.0 | <0.001 |
| BMI (kg/m^2^) | 25.5 ± 6.0 | 35.2 ± 4.8 | <0.001 |
| Waist (cm) | 84.3 ± 12.3 | 107.1 ± 7.2 | <0.001 |
| Systolic BP (mmHg) | 110.7 ± 12.4 | 128.9 ± 14.8 | <0.001 |
| Diastolic BP (mmHg) | 71.2 ± 8.8 | 84.4 ± 10.1 | <0.001 |
| Total cholesterol (mg/dl) | 176.9 ± 36.1 | 212.0 ± 22.2 | <0.001 |
| LDLc (mg/dl) | 109.8 ± 30.8 | 134.8 ± 12.3 | <0.001 |
| HDLc (mg/dl) | 52.5 ± 12.4 | 38.4 ± 7.4 | <0.001 |
| Triglycerides (mg/dl) | 73.1 ± 41.1 | 193.7 ± 100.2 | <0.001 |
| Apo AI (mg/dl) | 145.6 ± 23.7 | 125.8 ± 24.4 | 0.004 |
| Apo B (mg/dl) | 77.4 ± 23.8 | 116.3 ± 15.4 | <0.001 |
| hsCRP (mg/l) | 2.61 ± 2.44 | 4.83 ± 3.56 | 0.039 |
| Glucose (mg/dl) | 82.5 ± 8.5 | 87.4 ± 8.5 | 0.033 |
| Insulin (μIU/ml) | 11.2 ± 6.9 | 21.1 ± 11.4 | 0.003 |
| HOMA-IR | 2.33 ± 1.53 | 4.56 ± 2.54 | 0.003 |
| ROS production (AU) | 38.5 ± 22.4 | 57.9 ± 16.6 | 0.036 |
| MPO (pmol/l) | 14692.7 ± 7679.3 | 13253.6 ± 12044.6 | 0.654 |
| IL-6 (pg/ml) | 3.86 ± 3.63 | 3.17 ± 1.47 | 0.636 |
| TNF-a (pg/ml) | 4.92 ± 3.34 | 7.19 ± 3.45 | 0.153 |
| E-selectin (ng/ml) | 36.1 ± 17.6 | 42.9 ± 8.6 | 0.345 |
| ICAM-1 (ng/ml) | 116.6 ± 37.9 | 256.6 ± 190.6 | 0.009 |
| VCAM-1 (ng/ml) | 1137.6 ± 526.4 | 1114.9 ± 233.4 | 0.915 |

Data are expressed as mean ± SD. Statistical significance (p<0.05) was considered when compared by an umpaired Student’s t-test.
